# Aptasensor for the Detection of *Mycobacterium tuberculosis* in Sputum Utilising CFP10-ESAT6 Protein as a Selective Biomarker

**DOI:** 10.3390/nano11092446

**Published:** 2021-09-20

**Authors:** Umi Zulaikha Mohd Azmi, Nor Azah Yusof, Jaafar Abdullah, Faruq Mohammad, Shahrul Ainliah Alang Ahmad, Siti Suraiya, Nurul Hanun Ahmad Raston, Fatin Nabilah Mohd Faudzi, Sachin K. Khiste, Hamad A. Al-Lohedan

**Affiliations:** 1 Institute of Advanced Technology, Universiti Putra Malaysia, Serdang 43400, Selangor, Malaysia; umizulaikha.ika@gmail.com (U.Z.M.A.); jafar@upm.edu.my (J.A.); ainliah@upm.edu.my (S.A.A.A.); fatinnmf@upm.edu.my (F.N.M.F.); 2 Department of Chemistry, Faculty of Science, Universiti Putra Malaysia, Serdang 43400, Selangor, Malaysia; 3 Department of Chemistry, College of Science, King Saud University, P.O. Box 2455, Riyadh 11451, Saudi Arabia; hlohedan@ksu.edu.sa; 4 School of Medical Sciences, Universiti Sains Malaysia, Kubang Kerian, Kelantan 16150, Malaysia; ssuraiya@usm.my; 5 School of Biosciences and Biotechnology, Faculty of Science and Technology, Universiti Kebangsaan Malaysia, UKM Bangi 43600, Selangor, Malaysia; nurulhanun@ukm.edu.my; 6 Department of Medicine, Harvard Medical School, Boston, MA 02115, USA; skhiste@bwh.harvard.edu

**Keywords:** portable detection systems, electrochemical aptasensor, *Mycobacterium tuberculosis*, CFP10-ESAT6, differential pulse voltammetry (DPV), iron/gold magnetic nanoparticles

## Abstract

A portable electrochemical aptamer-antibody based sandwich biosensor has been designed and successfully developed using an aptamer bioreceptor immobilized onto a screen-printed electrode surface for *Mycobacterium tuberculosis* (*M. tuberculosis*) detection in clinical sputum samples. In the sensing strategy, a CFP10-ESAT6 binding aptamer was immobilized onto a graphene/polyaniline (GP/PANI)-modified gold working electrode by covalent binding via glutaraldehyde linkage. Upon interaction with the CFP10-ESAT6 antigen target, the aptamer will capture the target where the nano-labelled Fe_3_O_4_/Au MNPs conjugated antibody is used to complete the sandwich format and enhance the signal produced from the aptamer–antigen interaction. Using this strategy, the detection of CFP10-ESAT6 antigen was conducted in the concentration range of 5 to 500 ng/mL. From the analysis, the detection limit was found to be 1.5 ng/mL, thereby demonstrating the efficiency of the aptamer as a bioreceptor. The specificity study was carried out using bovine serum albumin (BSA), MPT64, and human serum, and the result demonstrated good specificity that is 7% higher than the antibody–antigen interaction reported in a previous study. The fabricated aptasensor for *M. tuberculosis* analysis shows good reproducibility with an relative standard deviation (RSD) of 2.5%. Further analysis of *M. tuberculosis* in sputum samples have shown good correlation with the culture method with 100% specificity and sensitivity, thus making the aptasensor a promising candidate for *M. tuberculosis* detection considering its high specificity and sensitivity with clinical samples.

## 1. Introduction

Biosensors play a vital role in a plethora of applications, especially in the medical and food industrires, which comprise of the detection and quantification of proteins in clinical and food samples. Biosensors are described as the analytical devices used to detect the presence or quantify the concentration of a biological analyte by transducing the biochemical reactions of a bioreceptor with a specific target analyte into an optical, thermal or electrical signal [1]. Among different types of biosensors, electrochemical biosensors are of particular interest because of the lower limit of detection, simplicity, and easily miniaturized characteristics [2,3,4].

Bioreceptors are one of the key factors for explicit biosensor performance. Aptamers are single-stranded DNA or RNA sequences (most recently, peptides) that are generally less than hundred bases long [5]. Since their discovery in the 1990s, aptamers have received extensive interest for their application in biosensor development as an alternative to antibodies, which act as bioreceptors [6]. In general, the aptamers have to be isolated from the pools of random nucleic acid sequences by systematic evolution of ligands by an exponential enrichment (SELEX) process [7,8]. To specifically bind to their target, aptamers must fold into particular three-dimensional structures. Aptamers have been synthetically designed against a wide variety of targets, from small human molecules and viral proteins to whole microorganisms [9]. As compared to antibodies, aptamers are relatively easy to produce at a low cost, have high affinity towards their target and are generally stable [6,10]. On the other hand, aptamers can be synthesized in a large quantity with high reproducibility and purity [11]. A number of aptamer-based sensors have been developed using different transducer techniques and have demonstrated their excellent performance, which validates the promising potential of aptasensors [12,13,14].

Tuberculosis (TB) is one of the most common causes of death for those with human immunodeficiency virus (HIV), and this is due to the *Mycobacterium tuberculosis* (*M. tuberculosis*) pathogen [15]. This contagious disease is classified as airborne because it can be transmitted to other people when the infected person spews the bacteria into the air by coughing, sneezing, or even talking [16]. This pathogen’s main route of infection is through the respiratory system and will further travel into the body towards the lungs. Other than the lungs, this bacterium can become active and spread to other parts of the body when the immune system becomes weak. Globally, the number of reported TB cases increased from 6.4 million in 2017 to 7.1 million in 2019 [17]. In Malaysia, the number of TB cases are high, with a current estimated incidence of 92 cases per 100,000 population during the year 2019 [17]. This is due to the high influx of illegal immigration, HIV, drug resistance, delayed diagnosis, high smoking rates, and diabetes [18,19,20,21]. Therefore, early and accurate detection of TB will be of great help to isolate patients and curb the spread of the disease.

The aptamer used in this study is specifically designed with a high affinity towards *M. tuberculosis* through the SELEX process. It is used as the bioreceptor immobilized on the working electrode surface to specifically detect various biomarkers of *M. tuberculosis* such as CFP10 [22], ESAT6 [23], CFP10-ESAT6 complex [9,13], Ag85A [24] and MPT64 [5,25]. In this study, CFP10-ESAT6 complex, an antigen secreted by *M. tuberculosis*, was chosen as a biomarker because of its better sensitivity compared to either CFP10 or ESAT6 antigen alone. Besides that, it is capable of avoiding false-positive results (better specificity) that usually happens due to a Bacillus Calmette–Guérin (BCG) vaccination [13]. Aptamer is widely known to be more selective than other commonly used antibody bioreceptors and this will increase the stability and robustness of the developed sensor.

Graphene (GP), in the form of a two-dimensional honeycomb, has a lot of useful properties such as a large surface area (2630 m^2^/g), good electrical conductivity (200,000 cm^2^/Vs) and good thermal conductivity of ~5000 Wm/K [26]. It has been commonly used in biosensors due to its biocompatibility and non-toxic nature at optimal concentrations [27,28]. Meanwhile, polyaniline (PANI) is a conductive polymer that has been widely used because of its easy preparation process and low cost. In electrochemical sensors, PANI is reported to have fast electron transfer and excellent electrochemical activity [29]. Therefore, the combination of GP and PANI could enhance the conductivity on the surface of the working electrode in our proposed electrochemical biosensors, thus enhancing the signal transduction during biomolecule immobilization and detection [30]. This GP/PANI combination forms nanoparticle clusters on the working electrode’s surface and hence offers a high surface area to volume ratio for biorecognition of molecules immobilized as compared to direct immobilization on the working electrode [31]. The optimum surface immobilization could also contribute to the high-performance detection of the disease.

Signal amplification via sandwich-type electrochemical antibodies or aptamer-based assays can be achieved by using labelling tracers such as enzymes and nanoparticles (NPs). This method is commonly used to obtain high detection sensitivity along with the improvements in the selectivity. This limits the crosstalk and interfering signal [32]. The various types of nanomaterials have been investigated as labels, particularly metal NPs due to their high stability compared to enzymatic signal tags [33,34]. Besides, they also have a high surface area to volume ratio that could anchor a large number of biomolecules, which then enables a much higher signal response [35].

In recent years, gold NPs (AuNPs) have been widely used as a label in biosensing technologies due to their advantages. Different detection schemes implementing AuNPs as labels have been reported, such as the direct electrooxidation of Au(III) and a two-step detection scheme. A two-step detection scheme involves the pre-oxidation of Au^0^ to Au(III) by dissolution in acidic medium or HBr/Br_2_ mixture followed by stripping voltammetry detection or the direct reduction of the released Au(III) to Au^0^. The determination of AuNPs labels using electrochemical oxidation in hydrochloric acid (HCl) is the most common method in electrochemical biosensors [34,36]. In this detection approach, the AuNPs undergo the electrochemical oxidation process in order to form AuCl_4_^−^, which is called the pre-oxidation step. Then, the AuCl_4_^−^ formed is immediately reduced to metallic gold (AuCl_4_^−^ + 3e^−^ → Au^0^ + 4Cl^−^) and measured by voltammetric techniques, usually by differential pulse voltammetry (DPV) or square wave voltammetry (SWV). However, the acidity of HCl used needs to be evaluated since the use of highly acidic solutions contributes to the denaturation of biological material when used in decentralized settings [37]. Meanwhile, the performance of AuNPs could be enhanced by combination with magnetic NPs (MNPs). A work by Freitas et al. has shown that MNPs help to easily separate the target analyte from large and complex samples using an external magnetic field as well as enhance the reaction signal produced due to the high catalytic activity of MNP [38,39].

In this study, we proposed a sandwich-type electrochemical aptasensor for the detection of *M. tuberculosis* with modifications on the screen-printed gold electrode (SPGE) using iron/gold MNPs (Fe_3_O_4_/Au MNPs) as a label, while the GP/PANI nanocomposite serves as a signal amplification layer. The DPV technique was used to detect the CFP10-ESAT6 antigen. It was measured by the direct electrooxidation of gold in a neutral medium to form Au(III) ions. As compared with other sensors used for *M. tuberculosis* detection, this aptasensor is more specific in its choice of target.

## 2. Materials and Methods

### 2.1. Reagents

The aptamer sequence used in this work is 5′-NH_2_-GCC TGT TGT GAG CCT CCT AAC CCC ATC TTA TAC GTA TAT GGA CTC ATC TCG ACC CCC GAT AGG CTT GGT ACA TGC TTA TTC TTG TCT CCC-3′. The aptamer was purchased from Integrated DNA Technologies (Coralville, Iowa, US). *M. tuberculosis* CFP10-ESAT6 antigen and polyclonal antibody (Ab) were obtained from Cusabio (Houston, TX, USA). Bovine serum albumin (BSA), potassium hexacyanoferrate (III) (K_3_[Fe(CN)_6_]), potassium chloride (KCl), 2-mercaptoethanol (ME), 12-mercaptododecanoic acid (MDDA), (3-aminopropyl)triethoxysilane (APTES) and glutaraldehyde were all acquired from Sigma-Aldrich (St. Louis, MO, USA). Ethanol and sodium hydroxide (NaOH) were purchased from HmbG Chemicals (Hamburg, Germany) and R&M Chemicals (Essex, UK), respectively. All chemicals are of standard qualitative analytical grade. The aqueous solutions were prepared using ultrapure water unless otherwise specified. All cyclic voltammetry (CV) measurements were performed in 1 mM K_3_[Fe(CN)_6_] with 50 mM KCl. Differential pulse voltammetry (DPV) measurements were carried out in 0.1 M phosphate-buffered saline (PBS) with pH 7.4. 0.5 M of sulphuric acid (Sigma, St. Louis, MO, USA) was used to activate the SPGE before further modification. Real samples (sputum) used for testing were collected by Hospital Universiti Sains Malaysia (HUSM), Kelantan, Malaysia.

### 2.2. Instrumentation

An Eco Chemie Autolab PGSTAT302 benchtop potentiostat with a NOVA 1.5 module (Metrohm, Utrecht, the Netherlands) was used for the electrochemical immunoassay analysis while a portable reader (DRP-DROPCAST, DropSens, Oviedo, Spain) was used for the on-site clinical sample analysis. A screen-printed gold electrode (SPGE) was purchased from DropSens, Spain. It consists of a working electrode (WE) and counter electrode (CE) that are made of gold ink, while the reference electrode is made of silver/silver chloride (Ag/AgCl). All of these electrodes were printed on a ceramic support. Other equipment used included circular dichroism (CD) (Jasco, Portland, OR, USA), X-ray diffraction (XRD) (X’Pert-MPD, PHILIPS, Amsterdam, Netherlands), high-resolution transmission electron microscopy (HR-TEM) (JEOL, Akishima, Japan), and field emission scanning electron microscopy (FESEM) (NOVA NANOSEM 230, FEI, Hillsboro, OR, USA).

### 2.3. Preparation of Fe_3_O_4_/Au MNPs-Labelled Antibodies

Iron oxide/gold magnetic nanoparticles (Fe_3_O_4_/Au MNPs) were firstly prepared according to the previous protocol [31]. About 10 mg of Fe_3_O_4_/Au MNPs was mixed with 2-mercaptoethanol and 12-mercaptododecanoic acid (ME-MDDA) (1 mM, 4:1) prior to conjugation with Ab and incubated in dark conditions for 24 h. The functionalized Fe_3_O_4_/Au MNPs was washed several times with ethanol and water before being dispersed in 0.1 M PBS at pH 7.4. Then, the functionalized Fe_3_O_4_/Au MNPs were incubated for 2 h with 25 µg/mL Ab followed by washing using PBS solution. Lastly, the bio-conjugated MNPs (Fe_3_O_4_/Au-Ab) were blocked from non-specific binding by incubation with 1% BSA (2 h) and washed to remove excess BSA several times using PBS solution.

### 2.4. Electrochemical Aptamer-Based Assay Detection Scheme

The development of aptasensor for CFP10-ESAT6 detection was conducted according to the previous study [9]; but with a different type of transducer. The WE surface of SPGE was electrochemically treated with 0.5 M H_2_SO_4_ using a CV technique (potential range: 0.0–1.6 V, scan rate 100 mV/s) followed by deposition of 4 µL of 1 mg/mL GP/PANI onto the WE surface. The GP/PANI-modified SPGE was dried under room temperature overnight. Then, it was washed with ethanol to remove the unbound GP/PANI, followed by drying at 70 °C for 30 min. The capture aptamer (CapApt) immobilization process on the electrode surface was conducted through a cross-linking reaction using glutaraldehyde. The modified SPGE was immersed in 1% glutaraldehyde for 30 min at 4 °C. After the reaction, the modified electrode was rinsed with deionized water and dried at 37 °C. Then, 6 µL CapApt (20 µg/mL) was dropped onto the electrode surface, left to react for an hour and washed using deionized water. The surface of electrode was blocked with 0.25% BSA for 1 h at 37 °C to avoid non-specific binding. After washing with PBS washing buffer, the prepared electrode was incubated with 4 µL of CFP10-ESAT6 antigen solution with different concentrations at 37 °C for 1 h. Finally, the prepared Fe_3_O_4_/Au-Ab buffer solution (4 µL) was dropped onto the electrode surface. After incubation for 40 min followed by washing, the electrode was ready for the practical measurements.

### 2.5. Optimization of Experimental Conditions for the Bioreceptor

The concentration and incubation time of CapApt immobilized on the GP/PANI-modified SPGE were optimized to obtain the maximum performance of the aptasensor. The concentration of CapApt was tested in the range of 5 to 100 µg/mL with a constant concentration of CFP10-ESAT6 (20 ng/mL) immobilized on the electrode. The binding of CapApt and CFP10-ESAT6 was evaluated at various times ranging from 30 to 150 min. The binding was monitored by measuring the change in the oxidation current using DPV in 0.1 M PBS (pH 7.4) solution as described above.

### 2.6. Detection Study

The CapApt/GP/PANI-modified SPGE surface was incubated in different concentrations (5 to 500 ng/mL) of CFP10-ESAT6 for 1 h, rinsed twice with PBS to remove unspecific adsorbed CFP10-ESAT6 molecules and the response was recorded with DPV taken in 0.1 M PBS (pH 7.4) at the potential range of 0.0 to 1.0 V.

### 2.7. Specificity and Reproducibility Studies

For specificity studies, the sensor was incubated with 20 ng/mL CFP10-ESAT6, 2.5 mg/mL BSA (widely distinct protein), 20 ng/mL MPT64 (secreted by *M. tuberculosis* as well) and 10× dilution human serum for 1 h. After washing using PBS, Fe_3_O_4_/Au-Ab was dropped on the surface of the electrode and incubated for 40 min to complete the assay. The current response of different analytes was analysed using DPV by immersing the prepared electrodes in 0.1 M PBS (pH 7.4) at a potential ranging from 0.0 to 1.0 V. Meanwhile, for the reproducibility study, five individual electrodes were prepared for 20 ng/mL CFP10-ESAT6 antigen detection. After incubating the antigen for 1 h, the electrode was further incubated with Fe_3_O_4_/Au-Ab to complete the assay. The DPV was measured to evaluate the reproducibility performance of the developed sensor.

### 2.8. Real Sample (Sputum) Analysis

**Figure 1:**
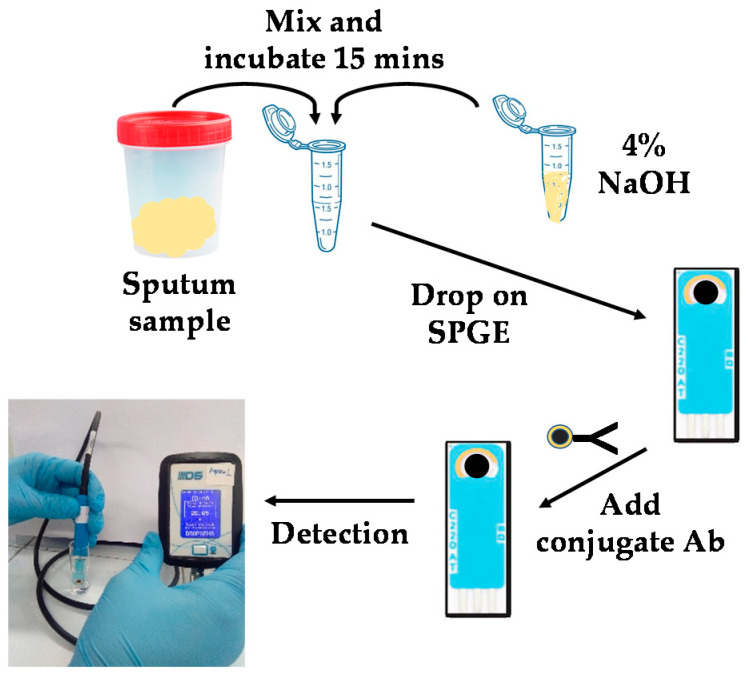
Sputum sample analysis for CFP10-ESAT6 antigen using portable readers. (i) The sample was decontaminated in 4% NaOH for 15 min and dropped onto the surface of modified electrode, (ii) Fe_3_O_4_/Au-Ab was dropped after the treated sample incubated on the capture aptamer (CapApt)/ graphene (GP)/polyaniline (PANI)/ screen-printed gold electrode (SPGE) surface for 40 min, and (iii) the prepared electrode was ready for measurements using portable reader.

Hospital Universiti Sains Malaysia (HUSM), Kelantan, Malaysia supplied 10 samples collected from patients. The patients were diagnosed with TB using the standard method of smear microscopy and a culture test to validate our developed electrochemical aptasensor. A culture test is a confirmation test for TB detection in hospitals in Malaysia. The results from the culture method will be used as a reference, while the performance of smear microscopy will be compared with the developed electrochemical aptasensor. The analysis was carried out at the laboratory of Medical Microbiology and Parasitology, HUSM. The clinical samples (sputum) were diluted in 4% NaOH prior to detection [40]. Decontaminated samples were dropped on the modified SPGE and incubated for 1 h; for the testing, at least 4 µL of decontaminated sample is required. After that, 4 µL of Fe_3_O_4_/Au-Ab was dropped onto the electrode and incubated for another 40 min. Finally, the electrode was immersed in 0.1 M PBS (pH 7.4) and ready for the measurements using a portable reader. The data displayed a value in the unit of µA. This whole process is shown in Figure 1.

## 3. Results and Discussion

### 3.1. Aptamer-Antibody Sandwich Interaction

**Figure 2:**
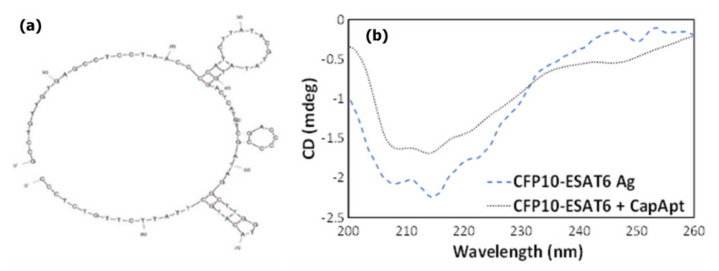
(**a**) Structure of capture aptamer for CFP10-ESAT6 antigen. (**b**) circular dichroism (CD) analysis indicates structural changes due to binding interaction between capture aptamer and CFP10-ESAT6 antigen.

As described in the experimental section, the aptamer was covalently immobilized on the working electrode surface to act as a capture probe for CFP10-ESAT6 protein. The selection of aptamer is based on a previous study [9], which developed the sequence of an aptamer for a CFP10-ESAT6 antigen. The structure of the aptamer is shown in Figure 2a. Beforehand, CD characterization was performed to confirm the binding and determine the conformational changes of CFP10-ESAT6 antigen and its aptamer. The CD is usually used for molecules such as nucleic acids and proteins containing chiral atoms. It measures differential absorption of left and right polarized light by the analytes [41,42]. As shown in Figure 2b, CFP10-ESAT6 antigen alone has a negative peak at 218 nm. Significant changes in the antigen structure were observed upon addition of CapApt to CFP10-ESAT6 antigen, indicating that aptamer folding occurred due to binding interactions.

**Figure 3:**
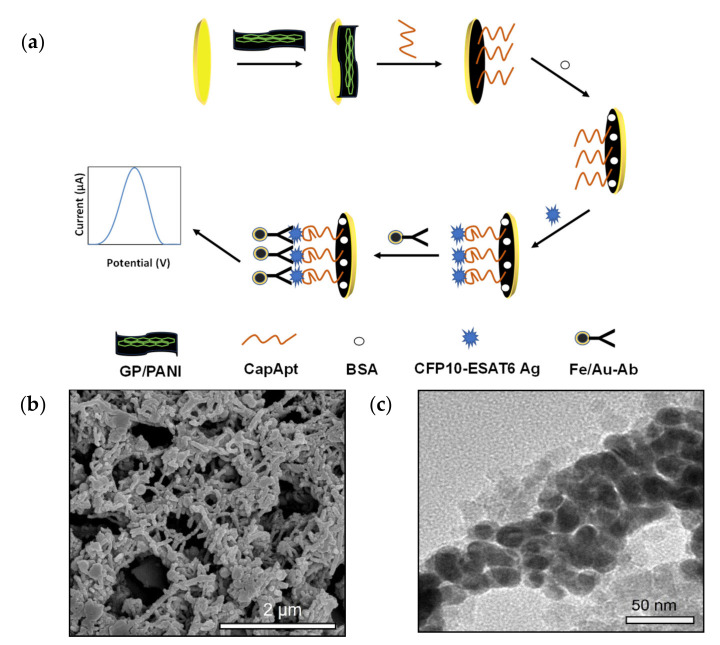
(**a**) Schematic flow of the electrochemical aptamer-based assay showing (i) modification of SPGE surface with GP/PANI, (ii) CapApt immobilization on GP/PANI-modified screen-printed gold electrode (SPGE), (iii) blocking by bovine serum albumin (BSA) to prevent non-specific binding, (iv) CFP10-ESAT6 antigen immobilization on CapApt/GP/PANI/SPGE surface, and (v) Fe_3_O_4_/Au-Ab immobilization on CFP10-ESAT6/CapApt/GP/PANI/SPGE surface. (**b**) GP/PANI nanocomposite structure and (**c**) Fe_3_O_4_/Au MNPs structure.

Figure 3a illustrates the different steps involved in the fabrication of a biosensor for the detection of CFP10-ESAT6 based on its specific bioreceptor by an aptamer–antibody assay. The covalent immobilization was conducted through a glutaraldehyde crosslinking reaction on the GP/PANI-modified SPGE surface. Figure 3b shows the field emission scanning electron microscope (FESEM) images of GP/PANI nanocomposite. The image of the GP/PANI nanocomposite showed that the GP sheets were mostly covered by PANI nanotubes and formed porous structures. This indicates that the presence of GP promoted the formation of agglomerated PANI nanofibers. The effect of the GP/PANI modification electrode surface has been elaborated in the Supplementary Materials (Section S2). Then, this step was followed by a blocking step with BSA to avoid non-specific adsorption on the transducer surface. Then, the Ab that was conjugated with Fe_3_O_4_/Au MNPs was deposited on the sensing platform to enhance the detection signal through sandwich type detection. Figure 3c shows the TEM images of the Fe_3_O_4_/Au MNPs that were used as nanoparticle labelling to increase the signal produced from the aptamer–antigen interaction. The average particles of Fe_3_O_4_/Au MNPs are around 35 nm, the molecular d-spacing was calculated from Image J software, which resulted in around 0.25 nm for the lighter part (iron core) and around 0.23 nm for the darker part (gold shell). The lattice distances measured for the shell correspond to the known Au lattice parameters for the (1 1 1) plane and those measured for the core match well the Fe_3_O_4_ lattice parameters for the (3 1 1) plane [43]. Preparation and crystalline structure characterization of this material have been further evaluated in the Supplementary Materials (Section S1). Finally, the biosensor was immersed in a PBS buffer solution of pH 7.4 and CFP10-ESAT6 was determined by differential pulse voltammetry of the generated AuCl_4_^−^ as the reaction product.

### 3.2. Optimization Studies of Aptasensor

**Figure 4:**
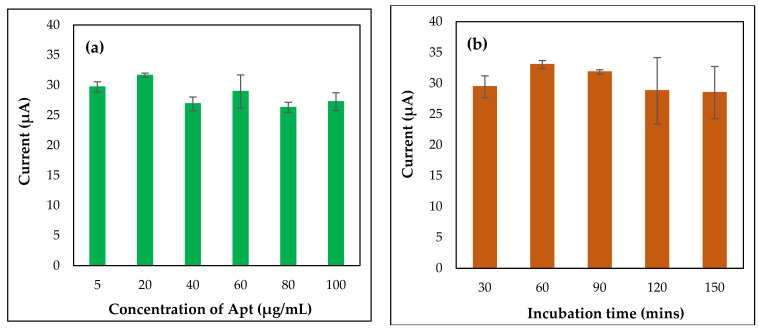
The effect of different concentrations (**a**) and immobilization time (**b**) of CapApt on the fabricated aptasensor in 0.1 M PBS at potential 0.0–1.0 V.

Optimization steps are very important and necessary to construct a good, stable, and reproducible biosensor. For this purpose, capture probe deposition parameters such as concentration of CapApt as well as incubation time of CapApt were examined. These conditions need to be optimized, as they influence the binding of the capturing probe with CFP10-ESAT6 antigen and affect the peak current of DPV. Different concentrations of CapApt (5, 20, 40, 60, 80, and 100 µg/mL) were studied by drop casting them onto GP/PANI-modified SPGE for 2 h so that the optimum concentration of CapApt bonding on the sensor could be determined. As seen in Figure 4a, the peak current increased with an increase in CapApt concentration (5 to 20 µg/mL). Nevertheless, the peak current started to decrease as the concentration increased from 40 to 100 µg/mL. This might be due to the over saturation of CapApt on the surface of GP/PANI-modified SPGE, which made CapApt less effective at binding with target antigens. Hence, the concentration of CapApt that was chosen to be used in further study is 20 µg/mL instead of 5 μg/mL. Besides, 20 μg/mL CapApt concentration provides better reproducibility as compared to 5 μg/mL. Figure 4b shows the effect of CapApt incubation time on peak current. From the results, the peak current reached the maximum peak at 60 min. Upon extending the incubation time, the current slowly dropped, which might be due to the instability of CapApt left at 37 °C for a longer time on the electrode surface. In general, the incubation time of 60 min is sufficient to record the detection of CFP10-ESAT6 antigen.

### 3.3. Limit of Detection (LOD), Specificity, and Reproducibility

**Figure 5:**
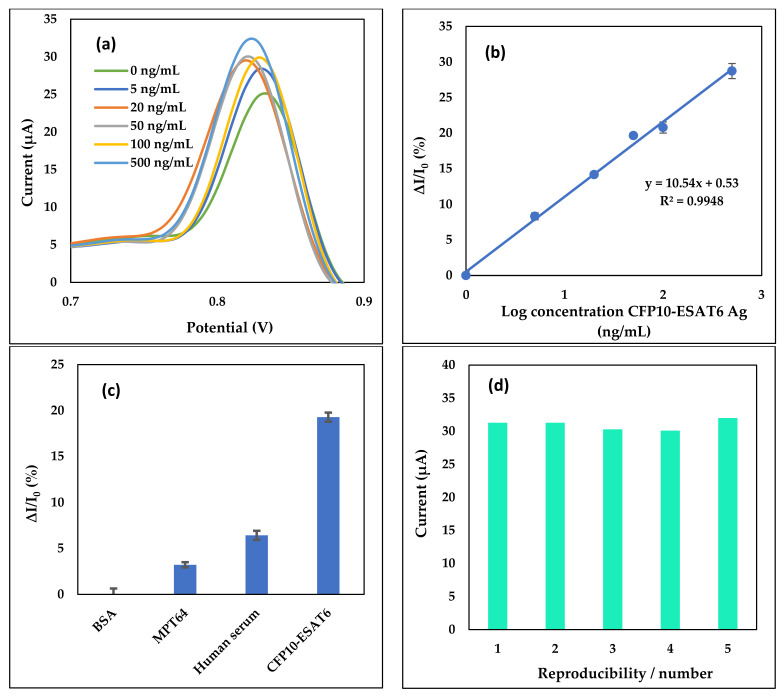
(**a**) differential pulse voltammetries (DPVs) of the aptasensor at different concentrations of CFP10-ESAT6 Ag, (**b**) the linear calibration plot of aptasensor in 0.1 M PBS (pH 7.4), (**c**) bar graph of specificity study using 2.5 mg/mL of BSA, 20 ng/mL of MPT64, 10× dilution of human serum and 20 ng/mL CFP10-ESAT6 using the developed aptasensor, and (**d**) bar graph of reproducibility study on aptasensor by using 20 ng/mL of CFP10-ESAT6 antigen in 0.1 M PBS at pH 7.4. Error bars show the standard deviations of replicate measurements.

Different concentrations of CFP10-ESAT6 antigen were detected by utilizing a specific constructed aptasensor. The analytical performance of our modified electrode was studied by using one of the most sensitive electrochemical analysis techniques, known as DPV. Figure 5a shows the current response of aptasensor upon interaction with the Fe_3_O_4_/Au MNPs conjugated Ab used for the detection of CFP10-ESAT6. It was shown that, with an increase in CFP10-ESAT6 concentration, the current response of Fe_3_O_4_/Au MNPs increased. This indicates an elevated interaction between Ab and antigen as the amount of Fe_3_O_4_/Au MNPs conjugated Ab increases.

A linear relationship between the analytical responses, Δ*I/I*_0_% signal and the logarithmic values of CFP10-ESAT6 concentration was found within the concentration of CFP10-ESAT6 ranging from 5–500 ng/mL as shown in Figure 5b. Δ*I/I*_0_% indicating; *I*_0_ = Peak current for blank (0 ng/mL CFP10-ESAT6), I = current response for certain concentration and Δ*I* = *I* – *I*_0_. The graph indicates an increase in sensor response signal with that of increased CFP10-ESAT6 concentration, which generated a linear positive slope. The calibration plot was fitted to a linear equation:

y = mx + c(1)

where y is the Δ*I/I*_0_% signal, m is the gradient slope and c is the y-intercept. The aptasensor exhibited a linear regression, expressed as y = 10.54x + 0.53 with a correlation coefficient of R^2^ = 0.9948. The LOD was obtained at 1.52 ng/mL of CFP10-ESAT6 concentration by using the 3σ/s calculation formula, where σ is the standard deviation of the blank and s is the slope of the calibration curve (Figure 5b).

The specificity study was conducted to prove the biosensor response is specific between the CFP10-ESAT6 antigen and the capturing probes immobilized on the electrodes. The study was conducted using 20 ng/mL CFP10-ESAT6, 2.5 mg/mL BSA, 20 ng/mL MPT64 and 10x dilution of human serum. As portrayed in Figure 5c, the highest peak current for the sensor was shown by CFP10-ESAT6 antigen, while the other pathogens were detected at a low peak current. This is because the pathogens other than CFP10-ESAT6 were not captured by CapApt. The aptasensor showed that the current difference between CFP10-ESAT6 with other pathogens is about 15% or more.

The reproducibility of the developed aptasensor was evaluated by means of relative standard deviation (RSD). Five independently modified electrodes were prepared for the detection of 20 ng/mL CFP10-ESAT6 antigen. From the bar chart in Figure 5d, the peak current obtained from 20 ng/mL CFP10-ESAT6 antigen detection on the five different modified aptasensor electrodes resulted in a current value in the range of 30–31 µA with an RSD of 2.5%. The consistency of signals recorded from the modified electrodes proved the reliability of the modification and stability of the detection process, thus showing its potential for mass production.

The analytical performance of the developed sensor for *M. tuberculosis* detection with other detection methods reported in the literature, together with the results, is shown in Table 1. Most of the detection was based on optical transduction techniques such as enzyme-linked immunosorbent assay (ELISA) [44,45], optical waveguide [46], fluorescence resonance energy transfer (FRET) [47], calorimetric magnetophoretic assay [48], and electrochemical sensor [49]. All of them except ELISA recorded a lower LOD when compared with our developed sensor, while our previous work recorded almost the same LOD. However, when compared with this work, the other methods still require multi-step amplification, are labour-intensive and time-consuming, and use heavy equipment, which obviously limit their use for point-of-care testing (POCT) for an extremely resource-limited environment. To the best of our knowledge, the electrochemical aptasensor that we developed in this work is capable of the direct detection of *M. tuberculosis* in sputum samples and can be implemented on POCT. Even though the LOD of our developed sensors was higher than several studies reported in Table 1, the LOD still falls in the physiological range of interest for *M. tuberculosis*, which is around 100 ng/mL in TB patients [20]. Besides that, our developed sensor can be easily miniaturized and transported for on-site detection during clinical application.

### 3.4. Clinical Sputum Samples Detection

**Figure 6:**
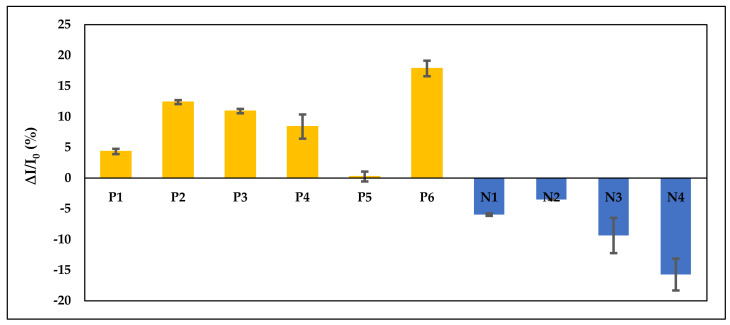
Current measured using portable reader from 10 clinical sputum samples immobilized on aptasensor. The results were identified as six positive (P) samples and four negative (N) samples, in accordance with the culture method.

A total of 10 sputum samples were analysed for clinical sample analysis. As the gold standard, results from the smear microscopy and culture method were used for comparison with our aptasensor reader. Each sample was identified as positive (P) or negative (N) according to the culture method (reference method). The culture method takes around 2–3 weeks to obtain the results, while smear microscopy takes around 15 min. The detection time using our portable reader is 2 h. From the reference method, it shows that there are six patients (out of 10) who were diagnosed positive and four were negative. From our portable reader, the results obtained were simplified in bar chart form as shown in Figure 6, where all of the positive samples show positive Δ*I/I*_0_% currents while the negative samples show negative Δ*I/I*_0_% currents.

Despite qualitative detection, this sandwich-type aptasensor is also able to quantify the concentration of CFP10-ESAT6 antigen in sputum samples, as shown in Table 2. By inserting the value of Δ*I/I*_0_% in a linear equation: y = 10.54 log x + 0.53, the concentration of CFP10-ESAT6 antigen were able to be determined.

The sensitivity and specificity of the smear microscopy method and our portable reader were compared by using the culture method as a reference and is shown in Table 3. The results obtained for the sensitivity and specificity of the smear microscopy and aptasensor reader was identical, which are 100%. From these findings, it was supported that our developed aptasensor is suitable for the detection of CFP10-ESAT6 antigen in sputum samples.

## 4. Conclusions

In this study, a sandwich type electrochemical aptasensor was designed for the detection of the *M. tuberculosis* antigen biomarker, CFP10-ESAT6. The SPGE was modified with GP/PANI for the immobilization of the capturing probe (CapApt). Meanwhile, Fe_3_O_4_/Au MNPs were used as a label to amplify the signal generation. The Fe_3_O_4_/Au MNPs were conjugated with primary antibodies to complete the sandwich format. The analytical performances of both sensors were successfully conducted by direct electrooxidation of AuNPs in PBS solution to form AuCl_4_^−^ due to the presence of chloride ions measured by DPV. It was found that the signal generated increased as the concentration of CFP10-ESAT6 antigen increased. The developed aptasensor was evaluated through the detection of tuberculosis in sputum samples and demonstrated 100% sensitivity and specificity. This suggests promising performance for the aptasensor and the commercialization of the aptasensor for TB and other diseases’ detection should be considered for early and effective illness prevention.

## Figures and Tables

**Table 1:** Limit of detection (LOD) of different detection method for *M. tuberculosis*.

Biomarkers	Biorecognition Elements	Detection Method	Detection Time	LOD	References
CFP10	Antibody	Plasmonic ELISA	>9 h	0.01 µg/mL	[44]
MPT64	Aptamer	ELISA	>2 h	0.5 mg/mL	[45]
ESAT6	Antibody	Waveguided-based optical biosensor	~2 h	100 pM	[46]
LAM				1 pM	
Ag85				0.5 pM	
Ag85B	Antibody	Fluorescence-based immunoassay	Not available	13.0 pg/mL	[47]
CFP10	Antibody	Calorimetric magnetophoretic immunoassay	~10 min	10 pg/mL	[48]
CFP10-ESAT6	Antibody	DPV electrochemical	~2 h	1.52 ng/ml	[49]
CFP10-ESAT6	Aptamer	DPV electrochemical	~2 h	1.5 ng/mL	Present work

**Table 2:** Comparison of various TB results from the culture method, smear microscopy, and portable aptasensor reader.

No	Concentration of CFP10-ESAT6 Ag (ng/mL)	Culture Method	AFB Direct Smear
P1	2.3	Positive	Positive
P2	13.3	Positive	Positive
P3	9.7	Positive	Positive
P4	5.6	Positive	Positive
P5	0.9	Positive	Positive
P6	44.1	Positive	Positive
N1		Negative	Negative
N2		Negative	Negative
N3		Negative	Negative
N4		Negative	Negative

**Table 3:** Sensitivity and specificity of smear microscopy and aptasensor methods used to identify *M. tuberculosis* in clinical sputum samples that were obtained from HUSM.

Methods	Positive Samples	Negative Samples	False-Negative Samples ^1^	False-Positive Samples ^2^	Total	Specificity (%)	Sensitivity (%)
Culture (Reference)	6	4			10		
Smear microscopy	6	4	0	0	10	100	100
Aptasensor reader	6	4	0	0	10	100	100

^1^ When a patient being diagnosed as negative but the bacteria are actually present, ^2^ When a person who is not actually sick is diagnosed as positive.

## Data Availability

The data presented in this study are available on request from the corresponding author.

## Supplementary Materials

The following are available online at https://www.mdpi.com/article/10.3390/nano11092446/s1, Figure S1: HR-TEM images of (a) Fe_3_O_4_ MNPs and (b) Fe_3_O_4_/Au MNPs dispersed in water and dried on copper grid, Figure S2: XRD patterns of Fe_3_O_4_ MNPs and Fe_3_O_4_/Au MNPs, Figure S3: FESEM images of (a) bare SPGE and (b) GP/PANI-modified SPGE, Figure S4: (a) Cyclic voltammograms and (b) Nyquist plots of bare SPGE, PANI-modified SPGE and GP/PANI-modified SPGE in 1 mM [Fe(CN)_6_]^3−/4−^ solution containing 50 mM KCl. Parameter for CV: Potential range: −0.4–0.6 V; Scan rate: 100 mV/s. Parameter for EIS: Frequency range: 0.01 Hz–100 kHz.
