# Supplementary material for: Aptasensor for the Detection of Mycobacterium tuberculosis in Sputum Utilising CFP10-ESAT6 Protein as a Selective Biomarker
Source: Nanomaterials (Basel). 2021 Sep 20;11(9):2446. doi: 10.3390/nano11092446 (PMC8470133; doi:10.3390/nano11092446)
Supplement: Supplementary file 1 [file nanomaterials-11-02446-s001.zip › nanomaterials-1367174-supplementary.pdf]

nanomaterials

MDPI

Article

Aptasensor for the detection of *Mycobacterium tuberculosis* in sputum utilising CFP10-ESAT6 protein as a selective biomarker

Umi Zulaikha Mohd Azmi[1], Nor Azah Yusof[1,2,*], Jaafar Abdullah[1,2], Faruq Mohammad[3*], Shahrul Ainliah Alang Ahmad[1,2], Siti Suraiya[4], Nurul Hanun Ahmad Raston[5], Fatin Nabilah Mohd Faudzi[1], Sachin K. Khiste[6], Hamad A. Al-Lohedan[3]

[1] Institute of Advanced Technology, Universiti Putra Malaysia, 43400 Serdang, Selangor, Malaysia; umizulaikha.ika@gmail.com, azahy@upm.edu.my, jafar@upm.edu.my, ainliah@upm.edu.my
[2] Department of Chemistry, Faculty of Science, Universiti Putra Malaysia, 43400 Serdang, Selangor, Malaysia; azahy@upm.edu.my, jafar@upm.edu.my, ainliah@upm.edu.my
[3] Department of Chemistry, College of Science, King Saud University, P.O. Box 2455, Riyadh, Kingdom of Saudi Arabia 11451; fmohammad@ksu.edu.sa; hlohedan@ksu.edu.sa
[4] School of Medical Sciences, Universiti Sains Malaysia, Kubang Kerian, 16150 Kelantan, Malaysia; ssuraiya@usm.my
[5] School of Biosciences and Biotechnology, Faculty of Science and Technology, Universiti Kebangsaan Malaysia, 43600 UKM Bangi, Selangor, Malaysia; nurulhanun@ukm.edu.my
[6] Department of Medicine, Harvard Medical School, Boston, MA 02115, USA; skhiste@bwh.harvard.edu
* Correspondence: azahy@upm.edu.my; fmohammad@ksu.edu.sa

1. Characterization of Fe_3O_4/Au MNPs

The preparation of Fe_3O_4/Au MNPs involved two steps; (i) the formation of Fe_3O_4 core and (ii) the coating of gold shell. The magnetite (Fe_3O_4) core was synthesized via co-precipitation method by adding an acidic solution containing Fe^{3+} and Fe^{2+} (with molar ratio 2:1) into a basic aqueous solution of NaOH. This method is simple, repeatable process, requires shorter time of reaction and easily understood mechanism. In addition, Fe_3O_4/Au MNPs could be dispersed in water as well. Equation (1) indicates the chemical reaction occurred for the formation of Fe_3O_4.

$$Fe^{2+} + 2Fe^{3+} + 8OH^{-} \rightarrow Fe_3O_4 + 4H_2O \quad (1)$$

Figure S1 shows TEM images of the Fe_3O_4 MNPs and Fe_3O_4/Au MNPs that were dispersed in deionized water. The average particles of Fe_3O_4 MNPs and Fe_3O_4/Au MNPs were ~10 nm and ~35 nm, respectively, which demonstrates the increment of size Fe_3O_4/Au MNPs was due to the coating of AuNPs on the Fe_3O_4 MNPs surface. As can be seen in **Figure S1 (a)**, Fe_3O_4 MNPs was agglomerated because there is no coating on the surface of Fe_3O_4 MNPs. After coating with AuNPs, good circular shapes were observed, and their sizes could be well measured individually (**Figure S1 (b)**). Meanwhile, the particles after gold coating seems to be much darker than Fe_3O_4 MNPs because gold shell has higher electron density than the iron core (Freitas et. al., 2014). The molecular d-spacing was calculated from Image J software, which resulting ~0.25 nm for lighter part (iron core) while ~0.23 nm for darker part (gold shell). The lattice distances measured for the shell correspond to the known Au lattice parameters for the (1 1 1) plane and those measured for the core match well the Fe_3O_4 lattice parameters for the (3 1 1) plane [1].

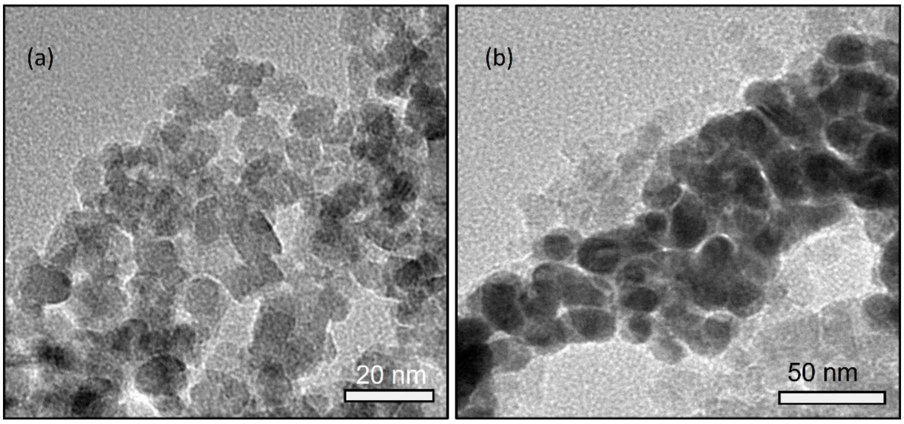

Figure S1. HR-TEM images of (a) Fe_3O_4 MNPs and (b) Fe_3O_4/Au MNPs dispersed in water and dried on copper grid.

The crystalline structures of pure magnetite Fe_3O_4 and after coated with Au shell were also confirmed by XRD. The XRD diffraction patterns of Fe_3O_4 MNPs and Fe_3O_4/Au MNPs are presented in **Figure S2**. The pure magnetite exhibited diffraction peaks (at 2θ = 30.5°, 35.5°, 43.4°, 57.2° and 62.7°), which were indexed to (2 2 0), (3 1 1), (4 0 0), (5 1 1) and (4 4 0) planes and corresponded well to the cubic structure of Fe_3O_4 (ICDD 01-08-1436). At the same time, Fe_3O_4/Au MNPs exhibited all the diffraction peaks of pure magnetite with additional peaks at 2θ = 38.1°, 44.4°, 64.5° and 77.4°, which could be respectively indexed to (1 1 1), (2 0 0), (2 2 0) and (3 1 1) planes of gold cubic phase (ICDD 01-089-3697). This information is corresponding to other results reported in the literature [1–3].

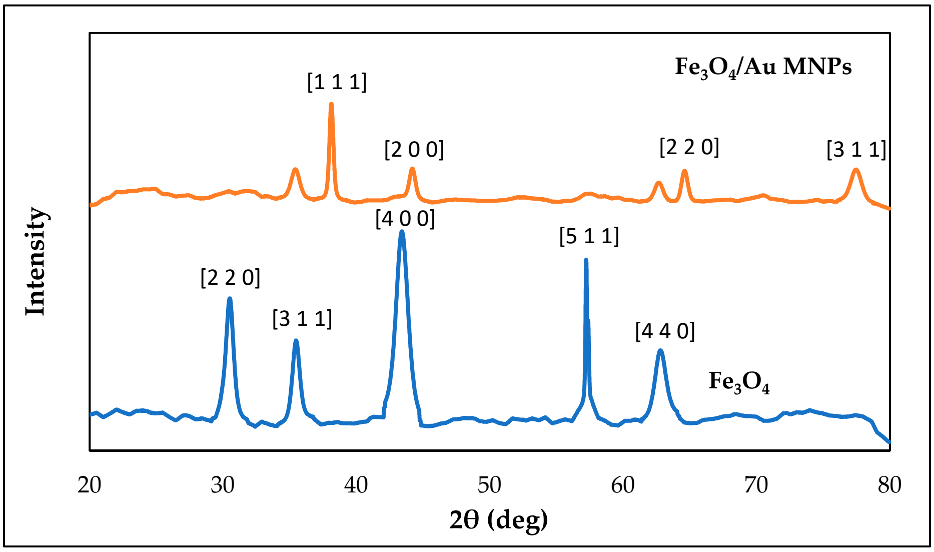

Figure S2. XRD patterns of Fe_3O_4 MNPs and Fe_3O_4/Au MNPs

2. Characterizations of GP/PANI-modified SPGE

The incorporation of GP/PANI nanocomposites on an electrode surface was carried out using a chemical modification strategy. The fabrication of GP/PANI-modified SPGE was described in detail in manuscript (Section 2.4). Firstly, the GP/PANI nanocomposites were dispersed in APTES because APTES was commonly used as an organosilane agent which acts as a dispersion agent and surface modifier for nanomaterials such as graphene [4]. Meanwhile, the bare electrode was treated with 0.5 M H_2SO_4 to generate a hydroxyl

group on the electrode surface prior to modification. To the best of our knowledge, there is no literature reported on the preparation of GP/PANI-functionalized electrode by drop casting of GP/PANI dispersed in APTES. In this reaction, the alkoxy groups of APTES bound to the hydroxyl group of the treated electrode.

The morphology of GP/PANI-modified SPGE surface was observed by FESEM. It was detected in **Figure S3** that the surface structure of GP/PANI-modified electrode was relatively rough and porous, compared to bare SPGE due to the formation of nanoparticle clusters on the electrode surface, which increased active surface area [5,6].

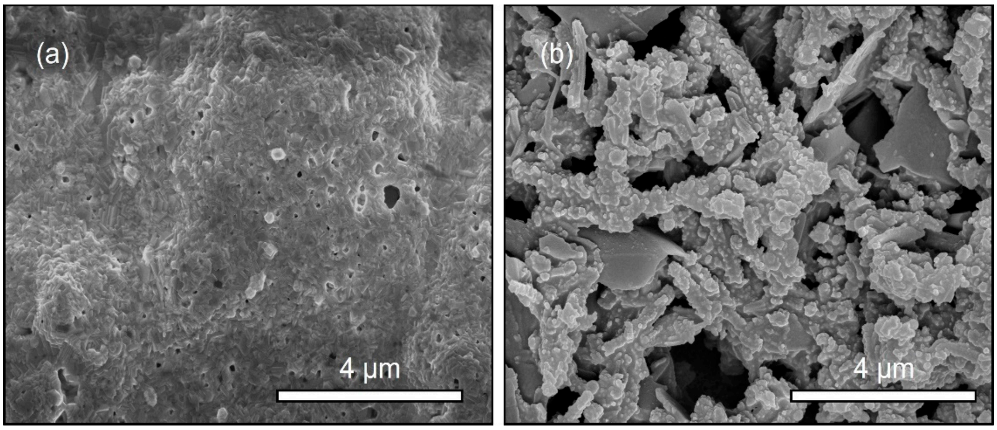

Figure S3. FESEM images of (a) bare SPGE and (b) GP/PANI-modified SPGE

The CV technique was used to evaluate the electrochemical properties of the modified electrode by utilizing a solution of potassium ferricyanide and ferrocyanide ($[Fe(CN)_6]^{3-/4-}$) as a redox probe. This is due to the fine reversibility and fast electrochemical reaction of ferricyanide ion ($[Fe(CN)_6]^{3-/4-}$) with electrode surface as reported in previous literature [7]. **Figure S4 (a)** depicts the CV response of different modified electrodes (bare SPGE, graphene (GP)-SPGE, polyaniline (PANI)-SPGE and GP/PANI-SPGE) in 1 mM $[Fe(CN)_6]^{3-/4-}$ containing 50 mM KCl in the potential window of -0.4 V to 0.6 V with a scan rate of 100 mV/s. The Nernst equation can be used to predict the Nernst equilibrium potential for the redox couple, E_{eq}. From the calculation, the potential for redox couple of $[Fe(CN)_6]^{3-/4-}$ was in the range of +0.3 V and +0.1 V. For bare SPGE, a pair of well-defined redox peaks corresponding to the electrochemical reaction of $[Fe(CN)_6]^{3-/4-}$ were observed with $\Delta E_P = 73$ mV. After modification of the electrode using GP, PANI and GP/PANI, the ΔE_P slightly increased, which were 95 mV, 105 mV and 132 mV, respectively. This is due to high barrier for electron transfer, thus, the electron transfer slowed down and more negative (positive) potentials are required to observe reduction (oxidation) reaction. However, the redox peak current (anodic and cathodic) was greatly enhanced after the modification of SPGE with GP/PANI nanocomposite due to the large surface area of GP/PANI (**Figure S4 (a)**).

An effective surface area of the electrodes can be calculated using the Randles-Sevcik equation (2): where I_{pa} is the oxidation peak current, n is the number of electron transfers (n=1), v is scan rate (V/s), D is the diffusion coefficient of $[Fe(CN)_6]^{3-/4-}$ solution with value of 7.6 x 10^{-6} cm^2s^{-1}, A is the effective surface area (cm^2) and C is the concentration of $[Fe(CN)_6]^{3-/4-}$ solution. A larger surface area can increase the efficiency of electroactive site for electrocatalytic reaction.

$$I_{pa} = (2.687 \times 10^5)\ n^{3/2} v^{1/2} D^{1/2} A C \quad (2)$$

where I_{pa} is the oxidation peak current, n is the number of electron transfers (n=1), v is scan rate (V/s), D is the diffusion coefficient of $[Fe(CN)_6]^{3-/4-}$ solution with value of 7.6 x

10^{-6} cm^2s^{-1}, *A* is the effective surface area (cm^2) and C is the concentration of $[Fe(CN)_6]^{3-/4-}$ solution. A larger surface area can increase the efficiency of electroactive site for electrocatalytic reaction.

In this work, the experiment was performed in 1 mM of $[Fe(CN)_6]^{3-/4-}$ containing 50 mM KCl solution at different scan rates ranging from 10 mV/s – 100 mV/s. By substituting constant parameter in the equation, the effective surface area can be determined through the slope value of I_{pa} versus $v^{1/2}$ graph. The effective surface derived from the Randles-Sevcik equation corresponded to the number of electroactive sites on the electrode surface, which is responsible for charge transfer to the species in solution. Based on the calculation, the effective surface area of bare SPGE, PANI-modified SPGE and GP/PANI-modified SPGE surface were found to be at 0.1012 cm^2, 0.5206 cm^2 and 0.5350 cm^2, respectively. It demonstrates GP/PANI-modified SPGE provided greater surface area compared to other electrodes. Moreover, GP/PANI-modified SPGE showed 5 folds higher than the bare SPGE surface. From these findings, it can be concluded that the GP/PANI-modified SPGE provided more active sites to immobilize the antibody and increased the acceleration electron transfer of ferricyanide ion on the rough electrode surface due to the presence of GP/PANI [8].

The resistivity behavior of modified electrodes was further examined through electrochemical impedance spectroscopy (EIS). **Figure S4 (b)** shows the Nyquist plots of different modified electrodes in 1 mM of $[Fe(CN)_6]^{3-/4-}$ containing 50 mM KCl as supporting electrolyte in a frequency range of 0.01 Hz to 100 kHz. The diameter of a semicircle in the high frequency region corresponds to the electron transfer resistance (R_{et}). In brief, the recorded R_{et} values are 387 Ω, 255 Ω and 170 Ω, which corresponding to bare SPGE, PANI-modified SPGE and GP/PANI-modified SPGE, respectively. The decreasing in R_{et} value of GP/PANI-SPGE shows that conductivity of the developed sensor was improved.

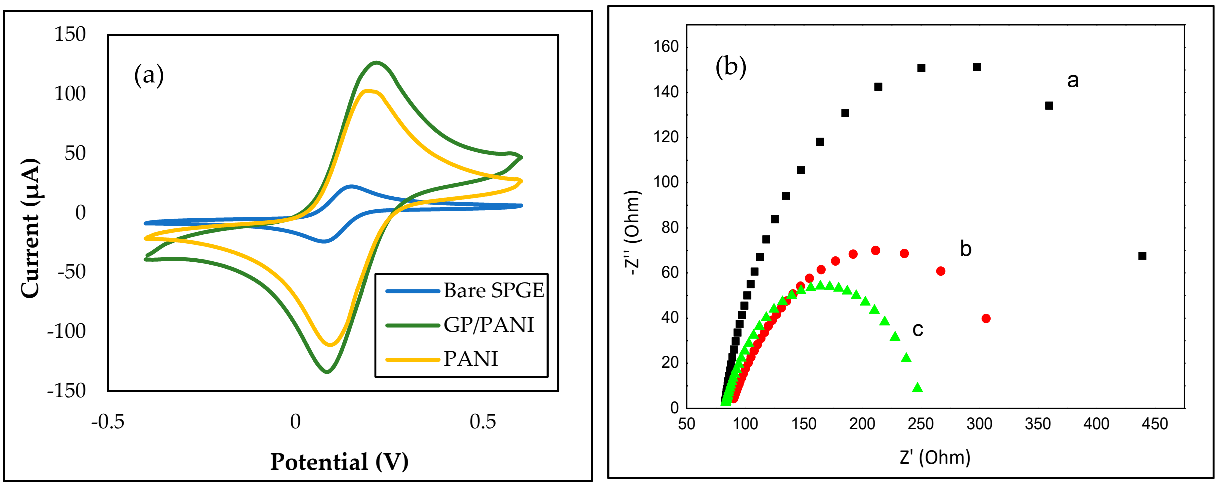

Figure S4. (a) Cyclic voltammograms and (b) Nyquist plots of bare SPGE, PANI-modified SPGE and GP/PANI-modified SPGE in 1 mM $[Fe(CN)_6]^{3-/4-}$ solution containing 50 mM KCl. Parameter for CV: Potential range: -0.4 – 0.6 V; Scan rate: 100 mV/s. Parameter for EIS: Frequency range: 0.01 Hz – 100 kHz.

References

1. Chauhan, R.; Basu, T. Functionalised Au Coated Iron Oxide Nanocomposites Based Reusable Immunosensor for AFB1 Detection. *J. Nanomater.* **2015**, *2015*, 1–16, doi:10.1155/2015/607268.
2. Cui, Y.-R.; Hong, C.; Zhou, Y.-L.; Li, Y.; Gao, X.-M.; Zhang, X.-X. Synthesis of orientedly bioconjugated core/shell Fe3O4@Au magnetic nanoparticles for cell separation. *Talanta* **2011**, *85*, 1246–1252, doi:10.1016/j.talanta.2011.05.010.
3. Zhou, H.; Lee, J.; Park, T.J.; Lee, S.J.; Park, J.Y.; Lee, J. Ultrasensitive DNA monitoring by Au-Fe 3O 4 nanocomplex. *Sensors Actuators B Chem.* **2012**, *163*, 224–232, doi:10.1016/j.snb.2012.01.040.

4. Zheng, D.; Vashist, S.K.; Al-Rubeaan, K.; Luong, J.H.T.; Sheu, F.S. Mediatorless amperometric glucose biosensing using 3-aminopropyltriethoxysilane-functionalized graphene. *Talanta* **2012**, *99*, 22–28, doi:10.1016/j.talanta.2012.05.014.
5. Mat Zaid, M.H.; Abdullah, J.; Yusof, N.A.; Sulaiman, Y.; Wasoh, H.; Md Noh, M.F.; Issa, R. PNA biosensor based on reduced graphene oxide/water soluble quantum dots for the detection of Mycobacterium tuberculosis. *Sensors Actuators B Chem.* **2017**, *241*, 1024–1034, doi:10.1016/j.snb.2016.10.045.
6. Abdul Rashid, J.I.; Yusof, N.A.; Abdullah, J.; Hashim, U.; Hajian, R. The utilization of SiNWs/AuNPs-modified indium tin oxide (ITO) in fabrication of electrochemical DNA sensor. *Mater. Sci. Eng. C* **2014**, *45*, 270–276, doi:10.1016/j.msec.2014.09.010.
7. Chan, K.F.; Lim, H.N.; Shams, N.; Jayabal, S.; Pandikumar, A.; Huang, N.M. Fabrication of graphene/gold-modified screen-printed electrode for detection of carcinoembryonic antigen. *Mater. Sci. Eng. C* **2016**, *58*, 666–674, doi:10.1016/j.msec.2015.09.010.
8. Mohamad, F.S.; Mat Zaid, M.H.; Abdullah, J.; Zawawi, R.M.; Hong Ngee, L.; Sulaiman, Y.; Rahman, N.A. Synthesis and characterization of polyaniline/graphene composite nanofiber and its application as an electrochemical DNA biosensor for the detection of Mycobacterium tuberculosis. *Sensors* **2017**, *17*, 1–17, doi:10.3390/s17122789.
